# Roles of autophagy in male reproductive development in plants

**DOI:** 10.3389/fpls.2014.00457

**Published:** 2014-09-15

**Authors:** Shigeru Hanamata, Takamitsu Kurusu, Kazuyuki Kuchitsu

**Affiliations:** ^1^Department of Applied Biological Science, Tokyo University of ScienceNoda, Japan; ^2^Department of Integrated Biosciences, University of TokyoKashiwa, Japan; ^3^School of Bioscience and Biotechnology, Tokyo University of TechnologyHachioji, Japan; ^4^Research Institute for Science and Technology, Tokyo University of ScienceNoda, Japan

**Keywords:** autophagic flux, male reproductive development, programmed cell death, tapetum, rice

## Abstract

Autophagy, a major catabolic pathway in eukaryotic cells, is essential in development, maintenance of cellular homeostasis, immunity and programmed cell death (PCD) in multicellular organisms. In plant cells, autophagy plays roles in recycling of proteins and metabolites including lipids, and is involved in many physiological processes such as abiotic and biotic stress responses. However, its roles during reproductive development had remained poorly understood. Quantitative live cell imaging techniques for the autophagic flux and genetic studies in several plant species have recently revealed significant roles of autophagy in developmental processes, regulation of PCD and lipid metabolism. We here review the novel roles of autophagic fluxes in plant cells, and discuss their possible significance in PCD and metabolic regulation, with particular focus on male reproductive development during the pollen maturation.

## INTRODUCTION

Reproductive development, both in animals and plants, is accompanied by drastic changes in metabolism for plentiful supply of nutrients and thus requires its appropriate regulation. In flowering plants, the anther exhibits a four-layered structure composed of the epidermis, endothecium, middle layer, and tapetum. Of these layers, the tapetum provides metabolites and nutrients to pollen grains, microspores, and the pollen coat during their development (Ariizumi and Toriyama, 2011). Tapetum contains triacylglycerol (TAG)-containing lipid bodies, which supply essential lipid components during pollen maturation (Li-Beisson et al., 2010; Murphy, 2012). Recent transcriptomic and bioinformatic analyses have suggested some factors that play roles in regulating lipid metabolism in the anther including a receptor-like kinase, proteases, cell wall-degrading enzymes, cytochrome P450 as well as lipid transfer proteins (Wang et al., 2008; Huang et al., 2009).

Autophagy is an evolutionarily conserved system for degradation and recycling of nutrients (Li and Vierstra, 2012). Intracellular components are enveloped by autophagosomal membranes and fused with the vacuoles/lysosomes, in which they are broken down by lytic enzymes. In animals, this process has recently been suggested to be involved in lipid droplet degradation, and defects in lipid autophagy (lipophagy) have been linked to important metabolic disorders such as fatty liver, obesity, and atherosclerosis (Dong and Czaja, 2011; Liu and Czaja, 2012). In many eukaryotes, autophagy is required for normal development, e.g., for dauer development in nematodes and preimplantation in mice (Tsukamoto et al., 2008; Melendez and Levine, 2009; Mizushima and Komatsu, 2011). In plants, autophagy has been suggested to be involved in seed development and germination, photomorphogenesis, chloroplast maturation, mineral nutrition, hormonal responses, pathogen resistance, stress protection, senescence, and fertile floret development under nutrient-limiting conditions (Ghiglione et al., 2008; Ishida et al., 2008; Chung et al., 2009). However, autophagy-defective *Arabidopsis* mutants exhibit normal life cycles, and little is known on the roles of autophagy during reproductive development in plants (Yoshimoto, 2012).

Programmed cell death (PCD), a genetically regulated form of cell suicide, plays vital roles in numerous physiological and developmental processes in multicellular organisms (Bozhkov and Lam, 2011; Fuchs and Steller, 2011; Teng et al., 2011). In plants, PCD is essential in various stress responses such as innate immunity against pathogen attack, and development including xylogenesis, pollen maturation, leaf senescence, seed germination, and embryogenesis (Pennell and Lamb, 1997). In angiosperms, cells in numerous reproductive organs undergo PCD during reproductive development, e.g., synergids, the anther tapetum, non-functional megaspores, the endosperm, the reproductive primordium, style transmitting tissues, abortive pollens, and antipodal cells (Greenberg, 1996; Pennell and Lamb, 1997; Wei et al., 2002). Homologs of many apoptosis-related genes in animals are not found in plants, and hence, it has been postulated that plants have evolved their own PCD mechanisms (Higaki et al., 2011; Li et al., 2011).

Autophagy is also involved in animal PCD (Shimizu et al., 2004; Tsujimoto and Shimizu, 2005), and has also been suggested to play critical roles in leaf senescence, a developmental PCD specific in plants (Ishida et al., 2008; Van Doorn and Woltering, 2010). Recent studies have shown that tapetal PCD, which plays extremely important roles in fertility, is regulated by a transcriptional network, reactive oxygen species (ROS) as well as activation of proteolytic enzymes in several plant species (Phan et al., 2011; Ma et al., 2012; Niu et al., 2013; Xie et al., 2014).

In this review, we describe temporal changes in autophagic flux that occur in tapetal cells and discuss the relationships between these changes and tapetal degeneration or metabolic regulation, with particular emphasis on plant anther development.

## ROLES OF AUTOPHAGY IN RICE ANTHER DEVELOPMENT AND POSSIBLE TAPETAL DEGRADATION DURING POLLEN MATURATION

The rice autophagy-defective mutants, *Osatg7* and *Osatg9*, show complete sporophytic male sterility and limited anther dehiscence under normal growth conditions, suggesting that autophagy is crucial in reproductive development in rice (Kurusu et al., 2014). Pollens of the autophagy-defective mutants are premature due to the significant defects in the anther during pollen maturation, while those of the heterozygous plants are normal and mature as the wild type, and the pollination of wild-type stigmas with the heterozygous pollens resulted in normal fertility. These findings indicate that parental tissue or organ defects cause the immature pollen phenotype displayed by the autophagy-defective mutants.

**FIGURE 1:**
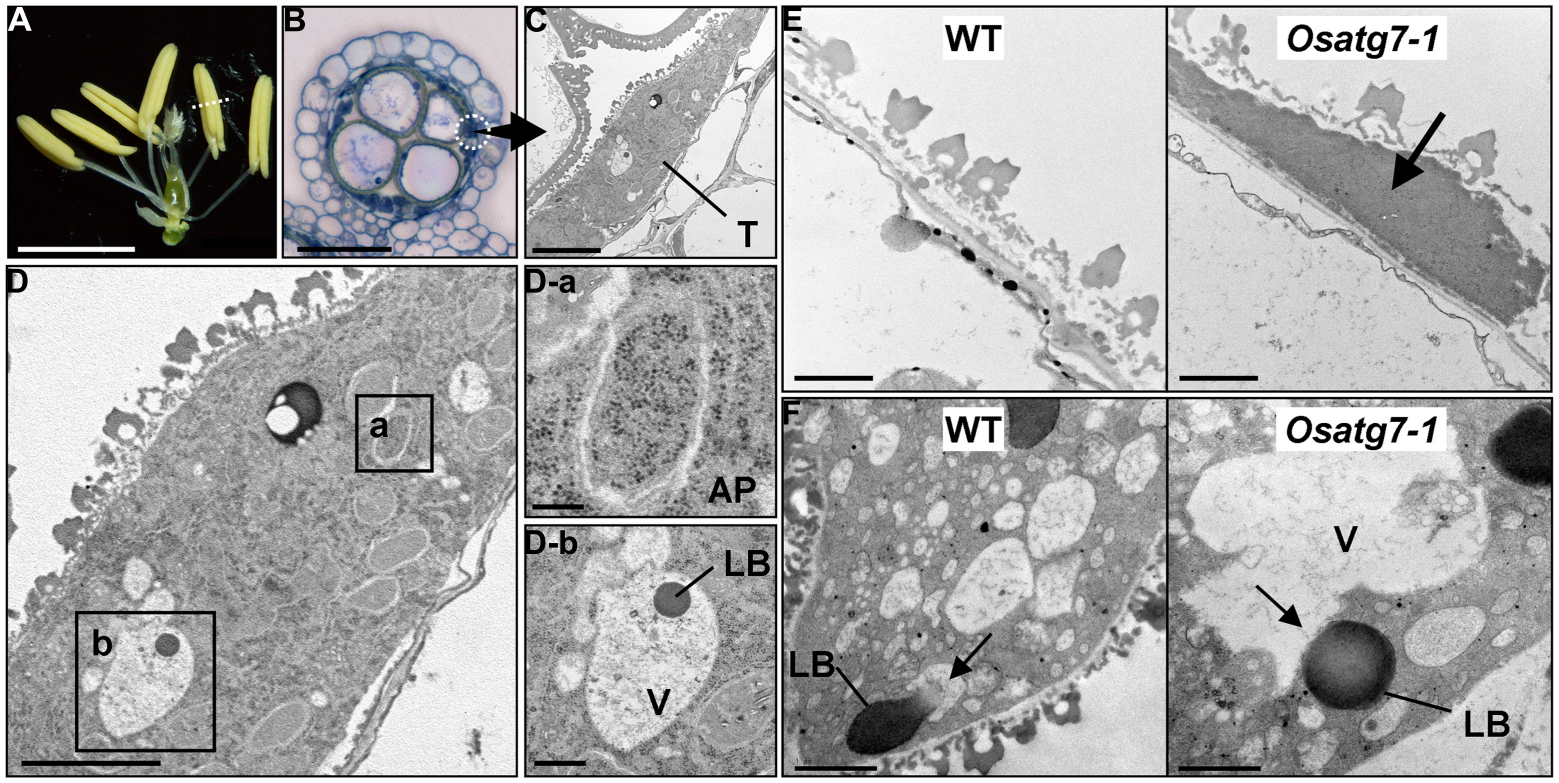
**Rice autophagy-defective mutants exhibit sporophytic male sterility, and autophagic degradation within tapetal cells is essential for postmeiotic anther development. (A)** Anthers in the wild-type at the uninucleate stage. Scale bar: 3 mm. **(B)** Transverse sections of wild-type anthers stained with hematoxylin at the uninucleate stage. Scale bar: 50 μm. **(C,D)** Autophagosome-like structures and lipid bodies enclosed within the vacuoles detected in postmeiotic tapetum cells during pollen development are depicted. Scale bars: 5 μm **(C,D)** and 1 μm **(D-a,b)**. **(E)** Potential role of autophagy in tapetal degradation and programmed cell death in rice. Tapetal ultrastructure of the wild-type and the *Osatg7-1* mutant at the flowering stage. Scale bar: 1 μm. Arrows indicate the tapetal cell layers.** (F)** Lipid bodies directly fuse with the vacuoles at the uninucleate stage in the rice tapetum. Scale bar: 1 μm. AP, autophagosome; LB, lipid body; V, vacuole; T, tapetum. Arrows indicate the vacuoles fused with lipid bodies in the wild-type (WT). Similar structure was not observed in the *Osatg7-1* mutant.

During pollen development, the tapetum provides metabolites and nutrients to pollen grains and microspores. Autophagosome-like structures, including dense globular bodies enclosed within the vacuoles, are detected in the tapetum during the uninucleate stage (Kurusu et al., 2014). On the other hand, the cytoplasm of the autophagy-defective mutants contained basically no autophagosome-like structures, indicating that autophagy is induced at the uninucleate stage in postmeiotic tapetum cells of rice and may be involved in the catabolism of intracellular components such as plastids and lipid bodies during pollen maturation (Kurusu et al., 2014; **Figures 1A–D**).

As pollens develop, the tapetum is broken down to provide nutrients, metabolites, and sporopollenin precursors to the developing microspores. Defects in tapetal degradation can result in the development of abnormal pollen coats and grains, leading to severe male sterility (Ku et al., 2003; Li et al., 2006; Zhang et al., 2008; Ariizumi and Toriyama, 2011). During tapetal degradation, which is tightly regulated, the characteristic features of PCD such as chromatin condensation, cell shrinkage, endoplasmic reticulum swelling, mitochondrial persistence (Rogers et al., 2005) and nuclear fragmentation (Wang et al., 1999; Vardar and Unal, 2012) are observed.

Appropriate temporal regulation of tapetal PCD is vital for normal pollen development. The signal initiating tapetal PCD has been suggested to be first produced during the tetrad stage (Kawanabe et al., 2006). Gibberellin controls tapetum degradation (Cheng et al., 2004; Aya et al., 2009). A delay in tapetal breakdown and a switch from PCD to necrosis in the tapeta were observed in an *ms1* mutant (Vizcay-Barrena and Wilson, 2006). Pollen wall deposition and the subsequent microspore degeneration failed in a rice mutant in which tapetal degeneration and PCD was retarded (Li et al., 2006, 2011; Zhang et al., 2008).

The rice *Osatg7-1* mutant was found to exhibit reduced anther dehiscence, which may contribute to its sterility (Kurusu et al., 2014). Furthermore, the *Osatg7-1* mutant exhibits a dense thin layer of tapetal tissue that came into contact with the orbicules, which is not observed in the wild-type even at the flowering stage (**Figure 1E**), suggesting that whilst the tapetal cell layer of the wild-type is completely degraded, its remnants remain in the mutant (Kurusu et al., 2014). As well as apoptosis, autophagy also plays a role in PCD and cell degeneration in animals (Shimizu et al., 2004; Tsujimoto and Shimizu, 2005).

Autophagic cell death typically involves the formation of double-membrane autophagosomes within the dying cells, which act to remove cell debris (Gump and Thorburn, 2011). Furthermore, a study examining metamorphosis in *Drosophila melanogaster* found that the destruction of the salivary glands and digestive tract during the latter process were controlled via significant increase in autophagic activity before and during the cell death (Melendez and Neufeld, 2008). Taken together, autophagy may contribute to tapetal breakdown in rice.

Proper timing of tapetal PCD is tightly controlled by an evolutionally conserved transcriptional network mediated by several key transcription factors (e.g., MYB, MADS families) in *Arabidopsis* and rice. Some proteolytic enzymes including cystein proteases, which play roles in PCD, are often targets of the tapetal transcriptional network (Li et al., 2006; Phan et al., 2011; Niu et al., 2013), suggesting possible involvement of the proteases in the execution of tapetal PCD.

Possible involvement of ROS production has also been suggested to play a role in tapetal PCD. Characteristic ROS accumulation is shown in rice anthers, which was abolished in *mads3* mutant, in which tapetal PCD occurs prematurely (Hu et al., 2011). NADPH oxidase/respiratory burst oxidase homolog (Rboh)-mediated ROS production has recently been suggested to be essential for tapetal PCD progression and pollen development in *Arabidopsis* (Xie et al., 2014). Expression of Rbohs is also regulated by the transcriptional network regulating the tapetal PCD (Xie et al., 2014). Autophagy-deficient mutants of *Arabidopsis* such as *atg5* have been shown to over-accumulate ROS in leaves (Yoshimoto et al., 2009). The potential role of autophagy in the regulation of the tapetal transcriptional network as well as ROS signaling is an important topic for future research.

Dynamic reorganization of the vacuoles mediated by actin microfilament has been suggested to play a critical role in executing various PCD in plants (Higaki et al., 2007, 2011). During the tetrad stage, abnormal vacuolization in the tapetum can lead to inappropriate tapetal PCD, resulting in male sterility (Wan et al., 2010). Tapetal vacuoles have been suggested to provide enzymes capable of degrading the tetrad wall, which are subsequently secreted into the anther locules (Wu and Yang, 2005). These findings indicate that tapetal vacuoles play significant roles in anther development and PCD. In the tapeta of *Lathyrus undulatus* L., the vacuolar membrane ruptures and the vacuole collapses at the vacuolated microspore stage, resulting in the release of hydrolytic enzymes and the subsequent destruction of cellular components (Vardar and Unal, 2012). Vacuolar processing enzyme (VPE) is expressed in *Arabidopsis* anther (Hatsugai et al., 2006), suggesting that VPE-mediated proteolysis may be involved in tapetal PCD. Dynamics of the vacuole and autophagy during tapetal PCD should be elucidated in various species in order to understand the molecular mechanisms responsible for autophagy-mediated PCD and its physiological significance in reproductive development in plants.

## ROLES OF AUTOPHAGY IN THE REGULATION OF LIPID METABOLISM AND NUTRIENT SUPPLY FROM THE TAPETUM TO DEVELOPING MICROSPORES

During pollen maturation, lipid bodies containing triacyl glycerols (TAGs) in the tapetum are necessary as a supplier of lipid components (Li-Beisson et al., 2010; Murphy, 2012). However, regulation of lipid metabolism including lipid remodeling remains largely unknown.

Lipophagy; i.e., the autophagic catabolism of lipids, is a selective form of autophagy targeting intracellular lipids. In animals, lipophagy is involved in the breakdown of lipid droplets (Dong and Czaja, 2011), and some important metabolic disorders such as atherosclerosis, fatty liver, and obesity have been found to be associated with defective lipophagy (Dong and Czaja, 2011; Liu and Czaja, 2012).

Lipid bodies enclosed in the vacuoles are detected in rice tapetum cells. Lipid body-like structures in the cytoplasm at the bicellular stage are more abundant in the autophagy-defective mutant than the wild type. A lipidomic analysis of the mutant anthers indicated impaired phosphatidylcholine (PC) editing and lipid desaturation during pollen maturation. These results suggest that in rice anthers, tapetal autophagy is involved in breakdown of lipid bodies and regulation of lipid metabolism, especially Lands cycle-mediated PC editing and desaturation, which affect pollen maturation including pollen coat formation (Kurusu et al., 2014). These findings highlight the significance of autophagy-mediated regulation of lipid metabolism in development. Autophagy is also involved in turnover of peroxisomes (Honig et al., 2012; Shibata et al., 2013; Yoshimoto et al., 2014), which play a role in the regulation lipid metabolism/turnover (Linka and Esser, 2012). The potential role of autophagy in the regulation of lipid metabolism is an important topic for future research.

Since autophagy-defective *Arabidopsis* mutants complete their own life cycles (Yoshimoto, 2012), autophagy may not play a critical role in the regulation of anther development in *Arabidopsis*. The most significant difference of the tapetum between dicots and monocots is that dicots have tapetosomes that possess ER-derived vesicles and lipid droplets for delivery to the pollen surface (Hsieh and Huang, 2007), while monocots do not form lipidic tapetosomes in tapetal cells. Moreover, many cereals including rice and a vast majority of other plants contain the secretory-type tapetum. Their tapetum produces the orbicules termed Ubisch bodies that mainly transport tapetum-derived sporopollenin precursors to developing microspores. However, no such Ubisch bodies have been identified in *Brassicaceae* including *Arabidopsis*. A family of lipid transfer proteins is one of the candidates for delivery of sporopollenin precursors from tapetum cells to the developing microspores (Ariizumi and Toriyama, 2011). It may suggest a critical difference in the development of lipidic components in the pollen grains between rice and *Arabidopsis*. In fact, the structures and components of pollen coat are quite different between cereals and *Brassicaceae* (Wilson and Zhang, 2009; Ariizumi and Toriyama, 2011). Detailed imaging analyses of various tapetum developmental stages under environmental stress conditions in the *Arabidopsis* mutants may clarify a novel mechanism for autophagy-mediated PCD and its physiological significance in *Arabidopsis*.

Several types of autophagy-related pathways have been observed in plant and yeast cells. In macroautophagy and the cytoplasm-to-vacuole transport (CVT) pathway, double-membrane structure in the cytoplasm fuses with the vacuole. *Arabidopsis* roots exhibit macroautophagy, in which autophagosomes directly fuse with the vacuoles (Merkulova et al., 2014). On the other hand, in cultured tobacco cells, treatment with E-64c induced the accumulation of autolysosome-like structures around the nucleus, suggesting an alternative autophagic pathway than the macroautophagy (Moriyasu and Ohsumi, 1996). Microautophagy involving invagination of the vacuolar membrane has also been observed (Toyooka and Matsuoka, 2006), however, its dynamics are largely unknown in most plant cells. These results imply that there exist multiple autophagic pathways specific to each cell type, species and tissue in plants.

In rice tapetal cells, lipid bodies directly fuse with the vacuoles at the uninucleate stage, which is distinct from macro- or microautophagy and dependent on *OsATG7* (**Figure 1F**). Further characterization of the dynamics of lipid bodies in the tapetum in various developmental stages in other plant species including *Arabidopsis* along with genetic analyses may reveal a novel function of autophagy related to lipid metabolism and its physiological significance during postmeiotic anther development.

## CONCLUDING REMARKS AND FUTURE PERSPECTIVES

The GFP-ATG8 fusion protein has been shown as a useful marker to monitor the whole process of autophagy in animals and fungi (Klionsky et al., 2007). A tandem fluorescent protein-tagged ATG8 (RFP-YFP-ATG8)-based non-invasive and semi-quantitative monitoring technique for autophagic flux has recently established in tobacco BY-2 cells (Hanamata et al., 2013). A further advantage of this method is that the autophagic activity of the cells can be quantitatively monitored by simply measuring the fluorescence of cell suspension without using inhibitors such as concanamycin A, a vacuolar H^+^-ATPase inhibitor. Moreover, turnover and degradation of an autophagy-specific cargo protein cytochrome *b*5 fused with the photoconvertible fluorescent protein Kikume Green–Red (KikGR) has recently been monitored as a useful marker for autophagic flux in tobacco BY-2 cells (Tasaki et al., 2014). These technical advances in combination with genetic analyses may be useful to characterize environmentally induced autophagy in various specific tissues including tapetum, and reveal novel aspects of autophagy in PCD in plants.

Autophagy is rapidly activated in response to various stimuli as well as developmental processes to induce dynamic reorganization of cytoplasmic components and is involved in the regulation of a wide range of physiological functions in plants. The duration, frequency, amplitude, and selectivity of autophagy seem to affect the specificity of autophagic signaling. Recent studies on ATG proteins have revealed their complex structures, diversity, and regulatory mechanisms, and the identification of ATG8-interacting proteins that recruit specific cargo to the enveloping phagophore sheds new light in the selectivity of autophagy *in planta*. For future studies, more information on autophagy-mediated signaling events including downstream effectors and signaling cascades is required to better understand their roles. In addition, genetic studies in many plant species and molecular characterization of their associated molecules are necessary to understand their functions.

## Conflict of Interest Statement

The authors declare that the research was conducted in the absence of any commercial or financial relationships that could be construed as a potential conflict of interest.
